# Staphylococcal Sepsis with Multiple Abscesses, Urinary Tract Infection, and Bilateral Renal Vein Thrombosis in a Patient with Uncontrolled Diabetes Mellitus

**DOI:** 10.1155/2012/357502

**Published:** 2012-10-11

**Authors:** Malik A. A. Khan, Jonathan Michael Hunter, Christopher Tan, Mostafa Seleem, Peter J. O. Stride

**Affiliations:** ^1^The School of Medicine, University of Queensland, Brisbane, QLD 4072, Australia; ^2^Department of Medicine, Redcliffe Hospital, Locked Bag One, Redcliffe, QLD 4020, Australia

## Abstract

We report a case of staphylococcal sepsis with vascular complications including peripheral emboli and renal vein thrombosis. Bilateral renal vein thrombosis has not been reported as a complication of *Staphylococcus aureus* (SA) axillary abscess. Uncontrolled diabetes was the only detected predisposing medical condition. The patient was treated successfully with incision and drainage of soft-tissue abscesses and intravenous antibiotic for six weeks and with anticoagulation for renal vein thrombosis.

## 1. Case Report

A 50-year-old male presented to our hospital with two weeks of malaise, polyuria, dysuria, and fever. Physical examination revealed a tender fluctuant left axillary mass, painful purpura over the distal phalanges of his toes, and no cardiac murmur. He had a history of poorly controlled type two diabetes mellitus (DM) (HBA1c of 9.5%–11.0% during the last twelve months). There was no preceding history of vascular access, urologic surgery, urinary tract obstruction, or urethral catheterisation. Previous medication only included metformin and gliclazide.

Intravenous flucloxacillin was commenced prior to collection of blood cultures, which subsequently failed to grow any microorganism. His diabetes was brought under control with insulin. The left axillary abscess was incised and drained and vancomycin was added to his therapy.

**Figure 1:**
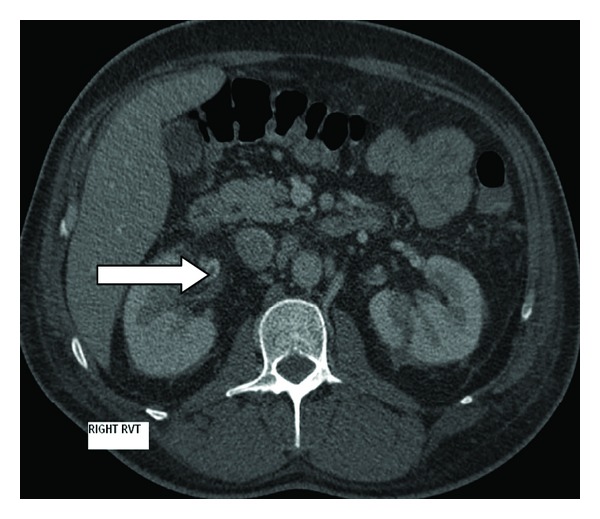


**Figure 2:**
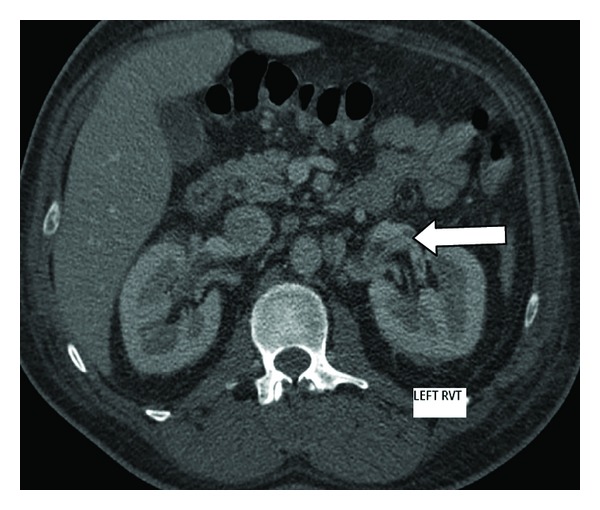


**Figure 3:**
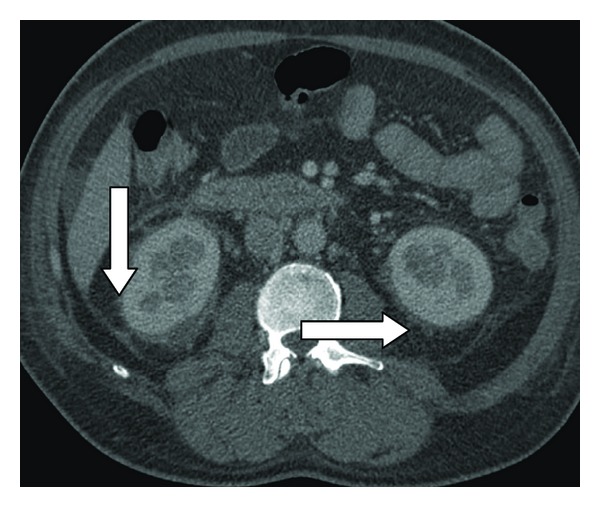
Perinephric stranding.

After 48 hours of therapy, he was still febrile. Mid stream urine and axillary pus both grew methicillin-sensitive SA (MSSA) and vancomycin was ceased. Computerised tomography (CT) of the chest and abdomen revealed bilateral renal vein thrombosis (Figure 1, right and Figure 2, left), perinephric fat stranding (Figure 3) and an abscess in the right thigh which was drained surgically. Cultures from the thigh abscess also grew MSSA.

Acute kidney injury occurred in setting of sepsis and renal vein thrombosis. Creatinine rose from 84 umol/L to 138 umol/L and remained stable until discharge. 

Technetium 99 Bone scan and transoesophageal echocardiography were inconclusive for evidence of primary or secondary focus of infection. He did not have personal or family history of venous thromboembolic disease. He was further assessed for hypercoaguable states, including the nephrotic syndrome. Twenty-four-hour urine contained 302 mg of protein. Thrombophilic screen was negative for factor V Leiden, lupus anticoagulant, anticardiolipin antibodies, protein C and protein S deficiency, and prothrombin gene mutation (2010G>A). 

**Figure 4:**
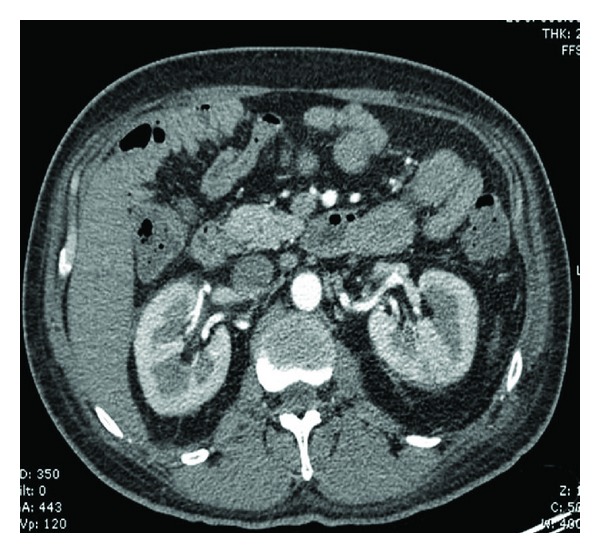
CT angiogram with no thrombus after 1 week of treatment.

Intravenous flucloxacillin was continued for six weeks. Monitoring of C-reactive protein and white cell count showed rapid resolution to normal ranges during treatment. Anticoagulation was also commenced with initial enoxaparin followed by warfarin. CT angiogram showed resolution of renal vein thrombosis after anticoagulation for one week (Figure 4).

## 2. Discussion

*Staphylococcus aureus* (SA) is a commensal in 11 to 32 percent of healthy individuals and 25 percent of hospital personnel [1]. Persistently colonized persons are at an increased risk of SA infection and risk is further increased by diabetes, intravenous drug use, haemodialysis as well as other immunocompromised states including HIV [2, 3]. Skin and related SA infections are responsible for 17 percent of cases with 14 percent 30-day mortality [4]. The incidence of staphylococcal endocarditis as a cause of staphylococcal sepsis is less than 5 percent but 30 day mortality is as high as 11.8 to 23.9 percent [4]. *Staphylococcus aureus* bacteraemia (SAB) and SA bacteriuria (SABU) can occur in patients following indwelling urinary catheterization (IDUC) and urologic surgery. Up to 19.5 percent, patients can develop concurrent SAB and SABU without IDUC and more likely to have MSSA with community onset [5]. Vertebral osteomyelitis and soft-tissue infections are also common causes of SAB associated with concurrent SABU [6]. Persistent SAB is associated with bacterial endocarditis (BE) in complicated SAB [5]. Our patient had SABU secondary to soft-tissue abscess with no evidence of persistent bacteraemia, osteomyelitis, or BE.

We conducted the literature review by using PubMed and Cochrane review using terms “staphylococcal sepsis and renal vein thrombosis.”

Our patient developed bilateral pyelonephritis complicated by renal vein thrombosis in the context of SA sepsis. This followed likely seeding from an axillary abscess and in the absence of a procoagulant state or nephrotic syndrome. Renal vein thrombosis is a well-known complication of the nephrotic syndrome particularly membranous glomerulo-nephropathy (MGN), with prevalence of 20–60% [7].

Renal vein thrombosis associated with pyelonephritis has become exceptionally rare since the advent of effective antibiotics [8]. Novelli et al. [9] reported right renal vein thrombosis following acute cholecystitis and pyelonephritis as with our case SA was the causative organism. Harris et al. [10] reported bilateral renal vein thrombosis from pyelonephritis but due to a *Klebsiella* species. Staphylococcal bacteraemia is associated with venous as well as arterial thrombosis, in the absence of prothrombotic disorders [11]. Multiple mechanisms involved in interaction of SA with various body tissues, including native and undamaged heart valves, bones, joints, and other solid organs. These interactions allow SA to seed from blood stream to other tissues. Staphylococci adhere to vascular endothelium and bind through adhesion receptor interaction [12]. A number of different adhesion molecules, including microbial surface component recognizing adhesive matrix molecules (MSCRAMM) such as protein A, clumping factor A (ClfA), fibronectin-binding proteins A (FnBPA), and fibronectin-binding protein B (FnBPB), allow SA to interact and invade human endothelium [12, 13].

MSCRAMM, ClfA, FnBPA, and FnBPB are not only responsible for binding and invasion of SA but also cause activation of platelet activation [14]. ClfA and FnBP bind fibrinogen allowing an interaction with platelet GPIIb/IIIa receptors and Fc*γ*RIIa [15, 16]. MSCRAMM-, ClfA-, FnBPA- and FnBPB-mediated binding, invasion by SA, and platelet activation via GPIIb/IIIa and Fc*γ*RIIa is likely a mechanism leading to thrombosis.

The detection of renal vein thrombosis complicating SA sepsis posed a difficult management dilemma in our patient. Prompt treatment was important in order to preserve renal parenchyma and prevention of thromboembolism. Medical therapy included volume resuscitation, anticoagulation with prolonged antibiotic therapy, and rigorous glycaemic control. Duration of anticoagulation is generally guided by the presence or absence of reversible cause or prothrombotic state. In our case, anticoagulation had been prescribed for three months. Treating septic renal vein thrombosis with anticoagulation is not based on any clear guideline or recommendation and could plausibly be associated with increased of risk of bleeding. Our patient had been followed up regularly and had no complication at the end of the three months with stable renal function (creatinine 122 umole/L). Catheter-guided thrombolysis can be successful in selected cases with acute RVT and acute renal failure [17]. Prognosis of RVT depends on multiple prognostic factors, including baseline renal function at the onset, speed of onset, and adequacy of collaterals, underlying aetiology as well as adequacy of treatment [18, 19]. 

## 3. Conclusion

To the best of our knowledge, this is the first paper on renal vein thrombosis complicating SA sepsis from an axillary abscess. Renal vein thrombosis should be suspected in cases of SA sepsis and unexpected acute kidney injury. Prompt management of the patient's infection and also anticoagulation lead to a successful recovery.
